# Anal fistula metastasis of rectal cancer after neoadjuvant therapy: a case report

**DOI:** 10.1186/s40792-022-01410-z

**Published:** 2022-03-31

**Authors:** Shota Fukai, Shingo Tsujinaka, Yasuyuki Miyakura, Natsumi Matsuzawa, Yuuri Hatsuzawa, Ryo Maemoto, Nao Kakizawa, Toshiki Rikiyama

**Affiliations:** Department of Surgery, Saitama Medical Center, Jichi Medical University, 1-847, Amanumacho, Omiya, Saitama-shi, Saitama, 330-8503 Japan

**Keywords:** Anal fistula, Rectal cancer, Anal metastasis, Implantation metastasis

## Abstract

**Background:**

Anal metastasis of colorectal cancer is very rare and may present synchronously or metachronously, regardless of pre-existing anal diseases. We report a case of anal fistula metastasis after completion of neoadjuvant therapy for rectal cancer, followed by surgical resection of the primary tumor and metastatic lesion.

**Case presentation:**

A 50-year-old man was diagnosed with rectal cancer located 5 cm from the anal verge, with a clinical stage of cT3N0M0. He denied any medical or surgical history, and physical examination revealed no perianal disease. He underwent preoperative chemoradiation therapy (CRT) consisting of a tegafur/gimeracil/oteracil potassium (S-1)-based regimen with 45 Gy of radiation. After completion of CRT, computed tomography (CT) revealed the primary tumor’s partial response, but a liver mass highly suggestive of metastasis was detected. This mass was later diagnosed as cavernous hemangioma 3 months after CRT initiation. He then underwent and completed six cycles of consolidation chemotherapy with a capecitabine-based regimen. Subsequent colonoscopy revealed the complete response of the primary tumor, but CT showed thickening of the edematous rectal wall. Therefore, we planned to perform low anterior resection as a radical surgery. However, he presented with persistent anal pain after the last chemotherapy, and magnetic resonance imaging revealed a high-intensity mass behind the anus, suggestive of an anal fistula. We considered the differential diagnosis of a benign anal fistula or implantation metastasis into the anal fistula. Fistulectomy was performed, and a pathological diagnosis of tubular adenocarcinoma, suggestive of implantation metastasis, was made. Thereafter, we performed laparoscopic abdominoperineal resection. Histopathological examination revealed well-differentiated adenocarcinoma, ypT2N0, with a grade 2 therapeutic effect. Subsequent immunohistochemistry of the resected anal fistula showed a CDX-2-positive, CK20-positive, CK7-negative, and GCDFP-15 negative tumor, with implantation metastasis. There was no cancer recurrence 21 months after the radical surgery.

**Conclusions:**

This is the first report of anal fistula metastasis after neoadjuvant therapy for rectal cancer in a patient without a previous history of anal disease. If an anal fistula is suspected during or after neoadjuvant therapy, physical and radiological assessment, differential diagnosis, and surgical intervention timing for fistula must be carefully discussed.

## Background

Anal metastasis originating from colorectal cancer is rare, with a reported incidence of 0.7%; this is believed to be caused by intraluminal and lymphovascular processes [1]. Recently, several studies have highlighted that metastasis can occur synchronously [2–7] or metachronously [8, 9]. Pre-existing anal diseases, such as anal fistula, fissure, or trauma, may be associated with the development of anal metastasis [2, 3, 6, 8]; however, it can also arise in normal squamous mucosa [4, 5, 7, 9].

Herein, we report a rare case of anal fistula metastasis after completion of neoadjuvant therapy for rectal cancer, followed by surgical resection of the primary tumor and metastatic lesion.

## Case presentation

**Fig. 1:**
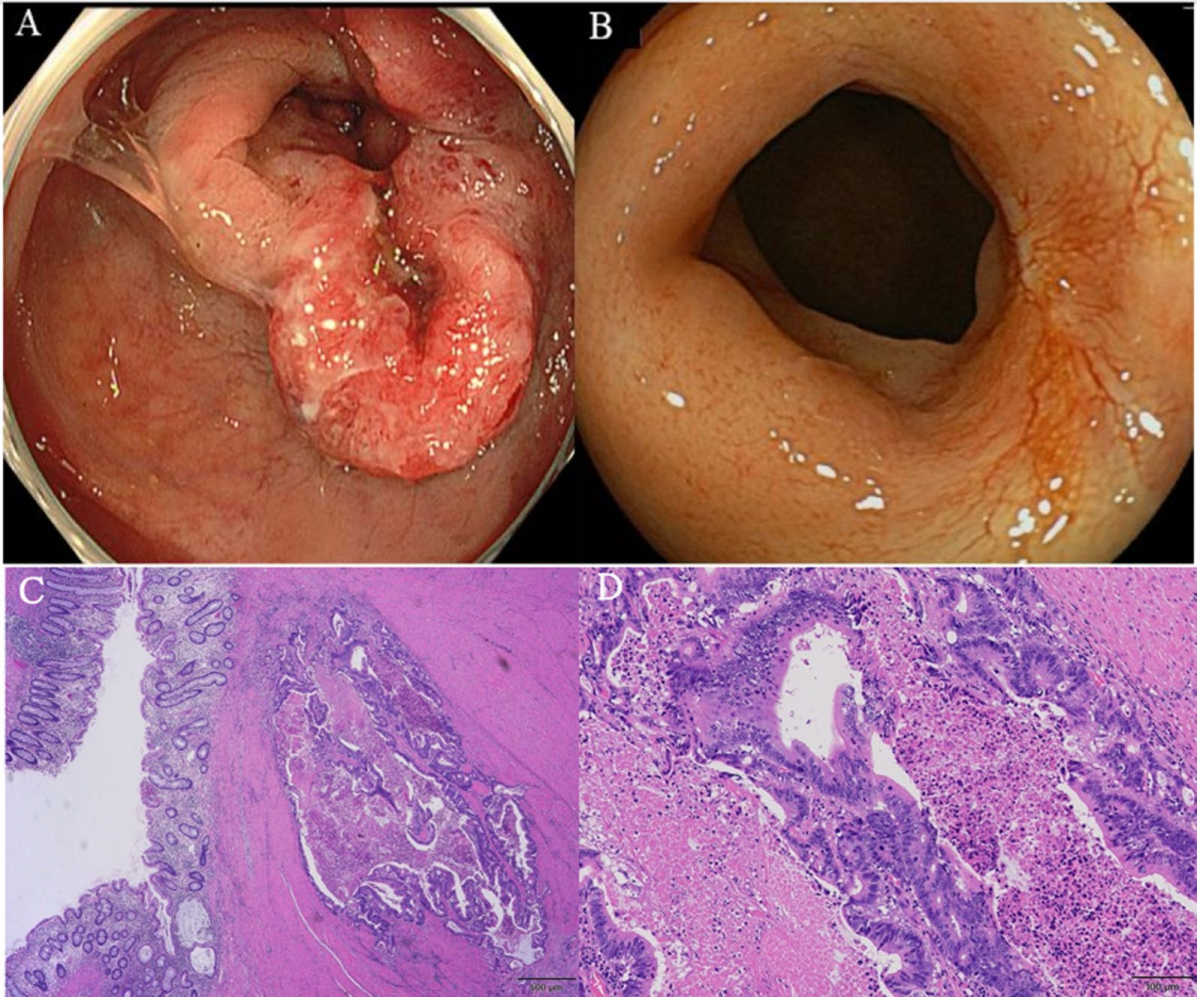
Preoperative colonoscopy. **A** Initial colonoscopy shows a rectal tumor below the peritoneal reflection 5 cm from the anal verge, occupying three quarters of the circumference. **B** Colonoscopy after chemoradiation therapy shows that the tumor responded clinically, and there is no evidence of the previous features, except for a flat white scar and telangiectasias. At the distal end of the tumor, the mucosa is reddish and swollen. Hematoxylin and eosin-stained sections (**C**, × 20. **D**, × 100.) show a well-differentiated adenocarcinoma with a partially tubular structure

A 50-year-old man presented with positive fecal occult blood test results. He denied any previous medical or surgical history. On digital examination, a solid tumor 5 cm from the anal verge on the posterior side of the rectum was palpated, and there were no anal fistulas or abscesses. Colonoscopy revealed a rectal tumor below the peritoneal reflection, occupying three-quarters of the circumference (Fig. 1A). A pathological diagnosis of the biopsy specimen indicated a well-to-moderately differentiated tubular adenocarcinoma. Computed tomography (CT) and magnetic resonance imaging (MRI) showed no significant regional lymphadenopathy or distant metastasis. Therefore, the clinical diagnosis was rectal cancer cT3N0M0, cStage IIa (UICC TNM classification 8th edition) [10].

We indicated preoperative chemoradiation therapy (CRT), comprising 45 Gy in 25 fractions; tegafur/gimeracil/oteracil potassium (S-1), 80 mg/m^2^/day at days 1–14 and 21–35 (2 cycles). The patient did not experience any significant adverse events. Four weeks after completion of CRT, a follow-up CT and MRI showed that the primary tumor had shrunk with a partial response as defined by the Japanese Society for Cancer of the Colon and Rectum (JSCCR) [11], but a small liver mass highly indicative of metastasis was also detected. This was later diagnosed as cavernous hemangioma using gadolinium ethoxybenzyl-diethylenetriaminepentaacetic acid-enhanced MRI 3 months after CRT initiation. We then indicated consolidation chemotherapy, consisting of capecitabine combined with oxaliplatin (XELOX regimen: intravenous oxaliplatin 130 mg/m^2^ [day 1] followed by oral capecitabine 1000 mg/m^2^ twice daily [day 1, evening to day 15, morning]). His tolerance to chemotherapy gradually declined due to peripheral sensory neuropathy (CTCAE Grade 1 to 2 [12]) after three cycles. As such, we excluded oxaliplatin and continued consolidation chemotherapy with three cycles of capecitabine alone (1000 mg/m^2^ twice daily [day 1, evening to day 15, morning]). Four weeks after completing consolidation chemotherapy, follow-up colonoscopy showed that the primary tumor achieved a complete response (Fig. 1B) based on the JSCCR guidelines [11]. Since a follow-up CT simultaneously showed thickening of the edematous rectal wall, we planned to perform low anterior resection as a radical surgery after neoadjuvant therapy.

**Fig. 2:**
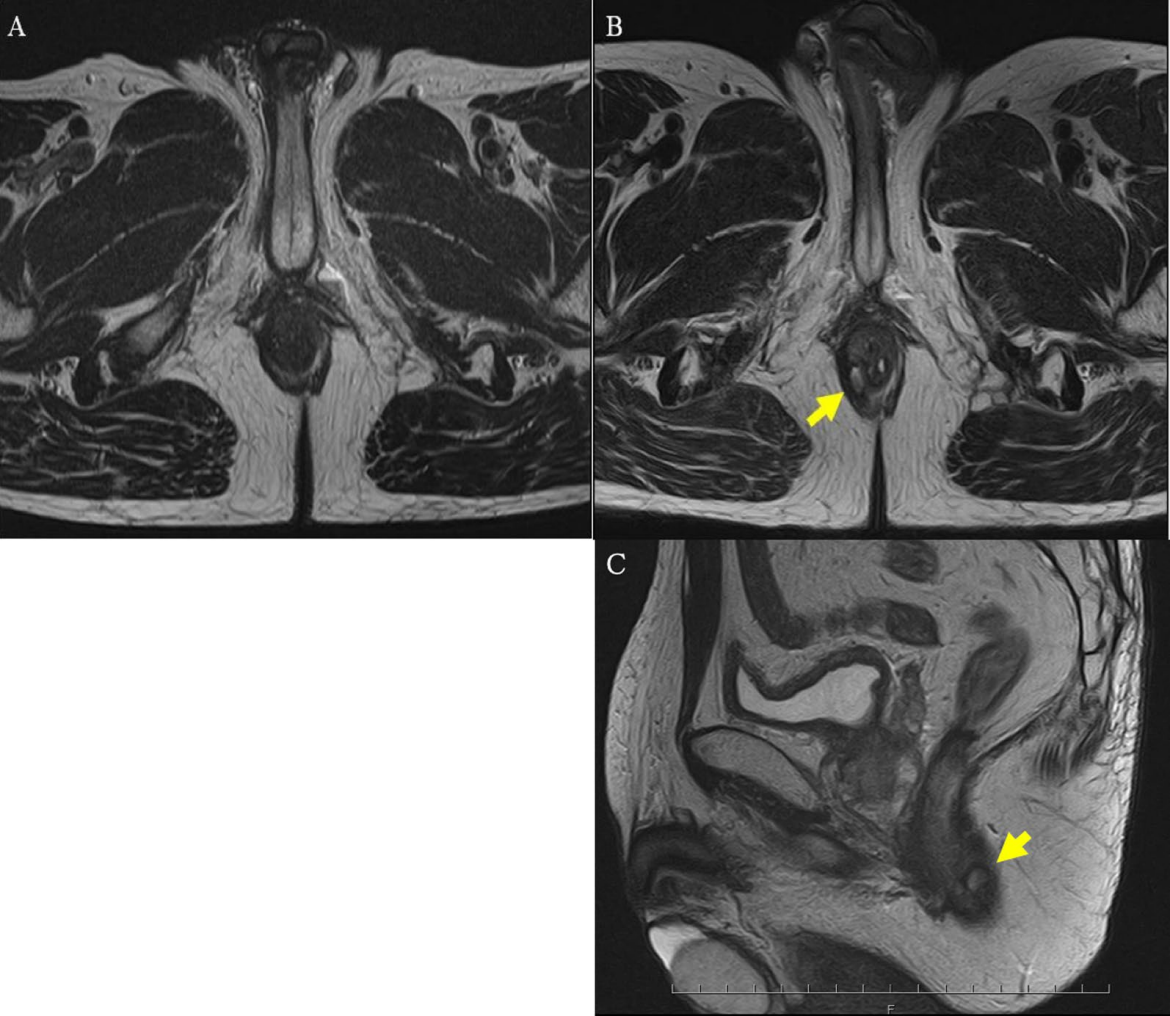
T2-weighted magnetic resonance imaging of the anal fistula. **A** Image obtained before preoperative chemoradiation therapy. No anal fistulas are observed. **B**, **C** Image shows a high-intensity anal fistula after chemoradiation therapy, located from the anal crypt to the skin behind the anus (arrows)

**Fig. 3:**
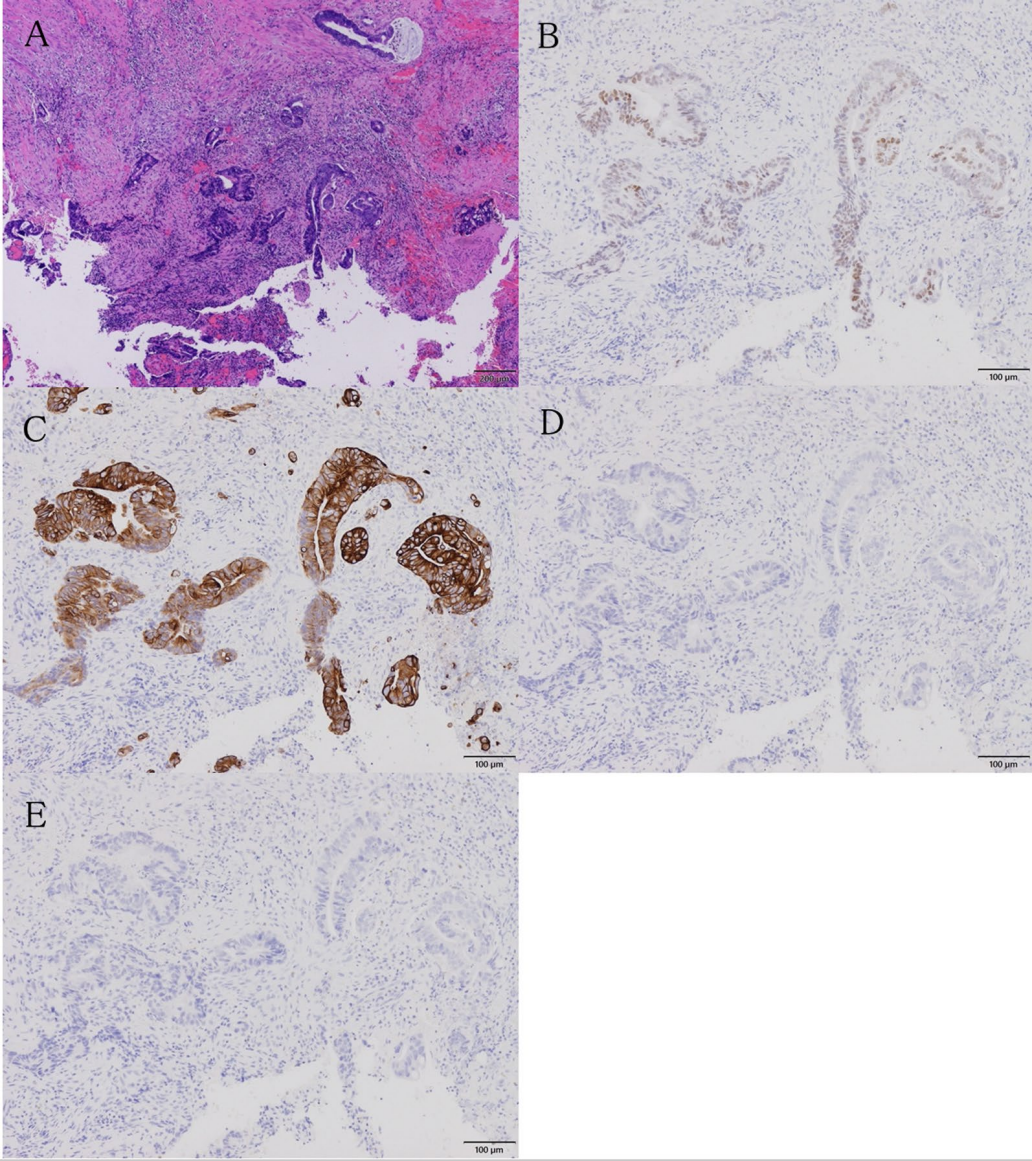
Pathological findings of the anal fistula. Pathological findings show an adenocarcinoma morphologically similar to rectal cancer and tumor cells infiltrating the proximal margin (**A**). On immunohistochemistry, the sample is CDX-2 positive (**B**), CK20-positive (**C**), CK7-negative (**D**), and GCDFP-15-negative (**E**)

However, he presented with persistent anal pain 2 weeks after the last chemotherapy session. Anorectal examination revealed tenderness and rigidity around the posterior side of the anus, which was distant from the primary tumor. MRI revealed a high-intensity mass behind the anus, suggestive of an anal fistula (Fig. 2). We considered the differential diagnosis of a benign anal fistula or implantation metastasis into the anal fistula. Coring-out fistulectomy was performed, and the resected fistula was pathologically diagnosed as tubular adenocarcinoma, which has similar morphological characteristics to rectal cancer (Fig. 3A). The resection margin was deemed positive because of the cauterized margin of cancer cells. This result suggested an implantation metastasis to the anal fistula. Therefore, instead of low anterior resection, abdominoperineal resection was performed to resect the primary rectal tumor and metastatic lesion simultaneously.

**Fig. 4:**
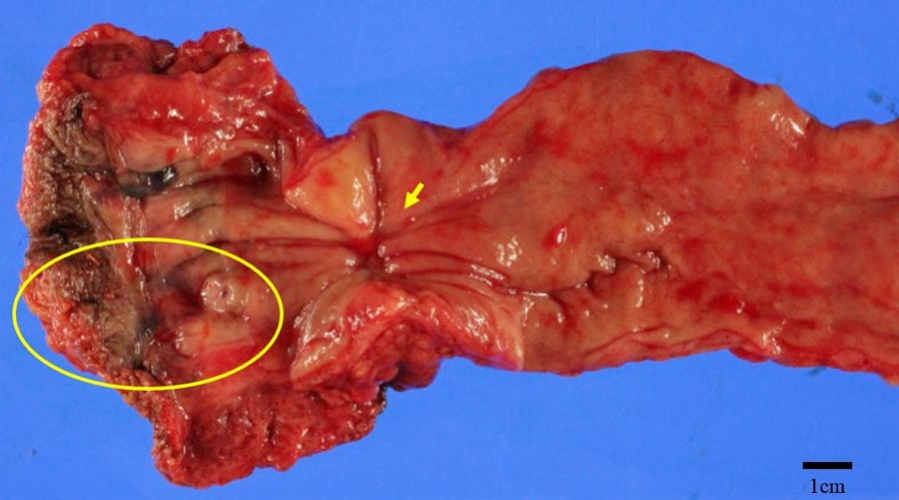
Gross and pathological findings of rectal cancer. The rectal lesion measures 10 × 10 mm and is classified as a type 2 tumor. Histopathologic evaluation showed an adenocarcinoma (tub1), ypT2, INFb, Ly0, V0, Pn0, pPM0, pDM0, ypN0 (arrow). The therapeutic effect grade was grade 2 [11]. There are no cancer remnants in the scar after anal fistula surgery (oval)

Twelve weeks after completing consolidation chemotherapy, we performed laparoscopic abdominoperineal resection. The postoperative course was complicated by a perineal abscess requiring percutaneous drainage. Otherwise, it was uneventful, and the patient was discharged on the 25^th^ postoperative day. Histopathological examination revealed a well-differentiated adenocarcinoma, ypT2, INFb, Ly0, V0, Pn0, pPM0, pDM0, ypN0 (Fig. 4). The therapeutic effect was grade 2 according to the definition of the JSCCR [11].

Subsequently, we investigated the origin of the tubular adenocarcinoma from the resected anal fistula by immunohistochemistry and found that it was CDX-2-positive, CK20-positive, CK7-negative, and GCDFP-15-negative (Fig. 3B–E). These findings implied that the anal fistula adenocarcinoma was consistent with metastasis and intraluminally implanted rectal cancer. The patient declined to undergo postoperative adjuvant chemotherapy. No recurrence was detected until his last follow-up 21 months after the surgery.

## Discussion

To the best of our knowledge, this is the first report of implantation metastasis to an anal fistula after neoadjuvant therapy for rectal cancer in a patient without a history of anal fistula. Persistent anal fistulas with recurrent inflammation are known risk factors for primary cancer [13]. Furthermore, when colorectal cancer spreads to the anal fistula, cancer cells shedding into the intestinal lumen are implanted in the injured mucosa [14]. Wound healing or an inflammatory response to a stimulus activates cancer cell growth, presumably resulting in cancer implantation into an anal fistula [7].

In most cases, the cause of anal fistula is considered to be non-specific cryptoglandular infection, and to a lesser extent, it is associated with inflammatory bowel disease, infections (such as actinomycosis, tuberculosis, lymphogranuloma venereum, human immunodeficiency virus), trauma, surgery, malignancy, and irradiation [15]. This patient did not have any signs of perianal disease, and the pretreatment images did not show any findings of an anal fistula. After completing neoadjuvant therapy, he developed persistent anal pain, and post-treatment images showed the presence of an anal fistula for the first time. Therefore, we believe that the anal fistula formed when he felt anal pain after completing consolidation chemotherapy, but the minimal histological changes in anal glands might have been initiated during neoadjuvant therapy.

In this patient, the pathological findings suggest that the anal fistula metastasis developed through a transluminal process during or after the neoadjuvant therapy. A follow-up colonoscopy 4 weeks after the completion of consolidation chemotherapy showed complete tumor remission. However, the patient presented with persistent anal pain 2 weeks after the last chemotherapy session: this was slightly earlier than the imaging studies that showed complete response. Therefore, the transluminal metastatic process of cancer cells might have begun before tumor response to neoadjuvant therapy.

Chronic radiation proctitis is characterized by mucosal fragility, pallor, spontaneous bleeding, telangiectasias, mucosal edema, strictures, fistulas, and ulceration [16]. Although we could not find any previous reports regarding anal fistula associated with neoadjuvant CRT, radiation therapy can lead to fistula formation as a late complication [17], and a previous report has shown that preoperative CRT is significantly associated with postoperative fistulous complications [18]. We assumed that radiation therapy might induce chronic injury to the corresponding anorectal mucosa, and malignant cells could have been delivered from the proximal large intestine and implanted into the anal fistula. Therefore, the implantation metastasis may have grown in the setting of chronic proctitis. We suggest the following three explanations for the etiology of anal fistula metastasis. First, long-term inflammation caused tumor hypoxia associated with increased radioresistance and a tendency to metastasize [19]. Second, although the location of anal fistula metastasis was within the range of irradiation, CRT and the following chemotherapy were not sufficiently effective to prevent transluminal implantation of cancer cells. Third, an anal fistula is an infectious condition; therefore, it may have become apparent when the patient was chronically immunocompromised by CRT or chemotherapy.

Recently, expectant management may be chosen for patients with rectal cancer, who showed a complete response after neoadjuvant therapy [20]. In our patient, colonoscopy revealed the complete response of the tumor after CRT and consolidation chemotherapy. Due to the presence of the anal fistula, expectant management was not offered. If an anal fistula is suspected after neoadjuvant therapy for rectal cancer in a patient without a history of anal fistula, the treatment strategy needs to be modified. The differential diagnosis of a benign anal fistula or implantation metastasis and the anatomical relationship between the primary tumor and the anal fistula determine the surgical approach, which includes abdominoperineal resection or sphincter-saving surgery [2–9]. In our patient, the primary tumor was originally located 5 cm from the anal verge; therefore, abdominoperineal resection was selected considering the sufficient resection margin of both primary and metastatic lesions. In addition, colorectal anastomosis would have been very proximal to the resection margin of anal fistula metastasis if sphincter-saving surgery had been performed.

When synchronous anal fistula metastasis is suspected, the timing of metastasectomy is controversial. Incisional or excisional biopsy before radical surgery is crucial for planning an appropriate treatment strategy for rectal cancer. However, the decision may be complicated, because several reports have suggested that anal metastasis can occur after injury to the mucosa or previous surgical site [2, 21, 22]. Currently, there is no established surgical management of anal fistula metastasis in rectal cancer. In our patient, abdominoperineal resection was performed as a radical surgery with complete tumor removal. Conversely, local resection with sphincter-preserving surgery and close surveillance can be an option for selected patients, and the surgical margin must be free of tumor cells to prevent local recurrence [5]. 

## Conclusions

This case highlights the importance of anorectal physical examination before, during, or after neoadjuvant therapy for rectal cancer, because implantation metastasis to the anal fistula can occur any time during treatment. Diagnosis of concomitant perianal disease guides the appropriate treatment strategy for rectal cancer. If an anal fistula is suspected, physical and radiological assessment, differential diagnosis, and surgical intervention timing for fistula must be thoroughly planned.

## Data Availability

The data sets supporting the findings and inferences of this case report are included in this article.

## Abbreviations

CRT: Chemoradiation therapy
CT: Computed tomography
S-1: Tegafur gimeracil, and oteracil potassium
MRI: Magnetic resonance imaging
